# Approaches Toward Building the Digital Enterprise and Sustainable Economic Development: The Moderating Role of Sustainability

**DOI:** 10.3389/fpsyg.2022.835602

**Published:** 2022-03-14

**Authors:** Jianhua Pei

**Affiliations:** School of Law and Economics, Changzhi University, Changzhi, China

**Keywords:** e-commerce, digitalization, digital marketing, economic development, sustainability

## Abstract

Achieving enduring economic development is the biggest challenge of today’s humanity. However, at present, there is a consensus that the digital economy plays a significant role in achieving sustainable economic development. The advancement of the digital enterprise is critical for obtaining socioeconomic stability and growth. In explanation, this study focuses on gaining economic progression through deploying the modern concept of digitalization. The study analyzes the mediating relationship of digital enterprise among e-commerce, digitalization, and digital marketing and their effect on China’s economic development while considering sustainability as a moderating variable. The study has used primary data collection techniques, and the study sample size is 400 respondents. The research has used SmartPLS software to measure the relationship through bootstrapping and algorithms. The study has found significant positive mediation of digital enterprise and moderation of sustainability between digital enterprise and economic development. This study suggests the theoretical and practical implications toward political stability, policymakers, and researcher perspective.

## Introduction

Nowadays, data and digital innovation are the main points of interest for most countries worldwide. The rapid development of e-commerce has immensely promoted the world’s economic advancement. E-commerce has modified the traditional business structure into an online business platform. It comprehensively strengthened the global economies to embrace innovative technologies, thereby achieving economic development (Tang and Wang, 2020). According to the firm viewpoint, the capacity to viably digitalization, digital marketing, and e-commerce has become a significant chance and test (Pan et al., 2020; Mohsin et al., 2021). The headway of innovation has helped global business. Many individuals utilize the internet to do everything, from leading examinations to buying items on the Web. The internet is significantly influencing practically all organizations.

Economic development might be accomplished through sustainability. Nations comprise adverse consequences without political stability and financial turn of events. Countries might have the chance to achieve economic development in a limited period while having political stability (Hutter et al., 2019). In an outline of the studies of economic development, Fukuyama required the improvement of a strategy that focuses on cultural support elements into current hypothetical and observational models which financial researchers are currently using.

Explicitly, the various employments of the internet by business substances incorporate the capacity to promote, create, or perform standard business capacities for economic development. Along these lines, many firms accept the digital enterprise for a significant number of their exercises with the moderation of sustainability. One effect for the digital enterprise is to heighten contest and produce benefits for buyers, with lower costs and more decisions (Konopczyński, 2014; Shah et al., 2021). The internet and Web-based business led to productivity upgrades, better resource usage, quicker an ideal opportunity to showcase, decreased complete request satisfaction times, and improved client support (Gordon, 2019; Ajaz et al., 2020).

Fundamentally, the digital enterprise is significantly growing freedom for business-to-business and business-to-shopper Web-based business exchanges across borders. For business-to-shopper exchanges, Web sets had created an upheaval in worldwide e-commerce: the individualization of trading. Innovation has extended the customer’s commercial center to an extraordinary degree (Di Nardo et al., 2020).

The reception of data innovation comparatively displays network externality achieved by more clients, with the subsequent advantages for the clients (Kinelski, 2020; Pervez et al., 2022). In recent years, various studies have thought about R&D as an intermediary variable for information capital while analyzing the connection between digitalization, e-commerce, and digital marketing on economic development. Alongside digital enterprise’s quick advancement, internet business innovations have become a significant information capital for working with a business.

Researchers have attempted to introduce various hypotheses to clarify this gap. As indicated by numerous hypotheses of development in the size of state-run administrations, the design and financial requirements change with the improvement of nations. Accordingly, the size of the government is impacted as well (Ojo et al., 2018). The advancement of a digital economy will significantly affect the practical improvement of an economy and society. The advancement of a digital economy would have a supporting impact on the progress of a local economy. Yet, the advancement of the advanced economy would achieve a gigantic advanced separation.

Thus, the worth added to yield at three levels of the undertaking, area, and nation will be expanded; eventually, it will prompt monetary development, work usefulness development, productivity, and the government assistance of the customer (Mastio et al., 2019; Khalil et al., 2021). Experimental examinations and hypotheses show that the connection between digital enterprise on economic development can be researched through three different ways: assuming the speculations of economic development have three variables, which include digitalization, digital marketing, and e-commerce, the impacts of these elements have been inspected by few business analysts.

Perhaps, in the wake of digitalization, the increasing debate about the economic progression of the countries has gained considerable height over the past few decades. At present, research shows that developing nations are still in the hope of achieving socioeconomic sustainability through digitalization (Jiao and Sun, 2021). In particular, technology is the prime economic driver that needs researchers’ attention. However, in this face of gaining technological and economic development, this study formulates the goal of examining the mediating role of digital enterprises (e.g., digitalization) in accelerating the country’s economic advancement.

The study’s objectives are to measure the relationship among e-commerce, digitalization, and digital marketing on economic development. Second, it measures the mediation effect of digital enterprise among cultural support, academic standards, rules, and regulations on economic growth. Third, it analyzes the moderating effect of sustainability between political stability and economic development.

The significance of the study is that political stability in the development interaction is conclusive and significant as far as China’s development is maintained. Digital enterprise has consequences for the monetary conduct of countries through the utility capacity on the interesting side, and it is additionally persuasive on the maker treatment on the sustainability. The connection among digital enterprise and economic development furthermore proficiency on the side of the economy is dictated by some integral elements that include association and the executive’s experience, authoritative part, and administrative part and interchanges structure as a result on the stockpile side of the economy, among different variables going into the capital, consequently prompting the improvement of the creative interaction through capital developing, propels in innovation, and the nature of the workforce.

## Literature Review

### E-Commerce and Economic Development

E-commerce refers to the utilization of the internet to manage deals nationally and internationally. Internet business has come to take on two significant jobs; first, as a most successful and effective course and aggregator of data, and second, as a possible instrument for the substitution of numerous financial exercises once performed inside a business undertaking by those that should be possible by outside providers that rival each other to execute these exercises (Kabango and Asa, 2015; Sarfraz et al., 2021). Finally, a few researchers have examined the connection between E-commerce and economic development. A significant number of these investigations have inferred a positive connection between e-commerce and economic development.

Furthermore, the examinations in the context of firms show that e-commerce can expand effectiveness. Hanim (2014) focused on information sources and the advancement of e-commerce and economic development, which announces that online business expanded benefits for firms and prompted the improvement of countries. Their discoveries showed that e-commerce was significant in reconciling the economic development framework.

In this paper, the focus has been put on the issue of whether economic development changes can support e-commerce business. The outcomes showed that essentially, no presence of the public authority in the field of online business could prompt economic development and increment the portion of e-commerce business devices in online business.

The studies found that e-commerce has impacted capital efficiency, whereas economic development displayed a bigger efficiency upgrading impact. E-commerce in developing effective development has been perceived as many economic examination communities have been created, which shows the significance of public interest in the public legislative issues (Ginting et al., 2020). From the above literature, the following hypothesis is stated:

H1: The significant relationship was found between e-commerce and economic development.

### Digitalization and Economic Development

Digitization and other innovative advancements decidedly affect the general creation cycle of any area. A study of Maiti and Kayal (2017) uncovers that the progression in the assembling area prompts its development and economic developmental areas. Vorontsovskiy (2020) studies whether there is any such connection between digitalization and economic development. Mosteanu (2020) observes that digitalization prompts more interest in labor and turn of economic development. Khan et al. (2015) look at emerging countries and track down the data correspondence of digitalization and its foundation; interchanges are the main economic development factors.

Myovella et al. (2020) propose that because of digitalization in business strategies, numerous non-tradable administrations become tradable these days through online platforms. Barinov et al. (2020) showed that digital improvement, strategies of assembling and worldwide qualities, and interest for administration with rising pay are the drivers for the worldwide exchange administrations of economic development. Afonasova et al. (2019) track down proof of the absence of online data for SMEs in emerging nations. Because of data unavailability in the digital industry and high observing expenses, organizations do not broaden funds simply based on economic models.

The study is one of the significant elements for every organization and country for digital platforms to improve and increase their GDP (Khalin et al., 2019). According to Frame and Woosley (2004), information transparency and economic development significantly impact digitalizing trading and financing.

Indeed, digitalization is a new buzzword that has transformed worldwide businesses. With the blessing of digitalization, more products had offered to customers. Through introducing new digital channels, conventional modes of business practices have changed, thereby increasing the sales of the businesses. In particular, the company’s prosperity largely depends on digitalization. Given the articulation, the study states that digitalization intensified the economic impact (e.g., productivity, efficiency, sales) (Kohtamäki et al., 2020), thus encouraging enterprises to adopt digital technologies as the prime driver of economic prosperity. Low productivity, low capitalization, resources, and high hazard are likewise the purposes behind organizations not implementing technological advancements. It is a critical variable in economic development because of functional contrasts between organizations and digitalization. Large organizations enjoy similar conditional benefits whereas small organizations benefit from implementing digitalization. From the above literature, the following hypothesis is stated:

H2: The significant relationship was found between digitalization standards and economic development.

### Digital Marketing and Economic Development

The previous studies showed digital marketing and its effect on economic development. In the new era of advancement, the cutting-edge society faces cardinal changes in all action circles. Digital marketing strategies have extraordinarily expanded the data field of individuals and researchers, which diminishes the expense of looking and handling data. The new development and rules of digital marketing have requested changes in the economy and their development, which has prompted the development of the expression “digital economy.” In view of the digital economy hypothesis, the idea of “digital marketing” has turned into a significant development of the economy (Teixeira and Piechota, 2019).

The primary motivation behind the logical article is the viable structure of promoting in the period of the digital economy. To accomplish this objective, the studies were recognized: an examination of brilliant advertising in the period of digital economic development — concentrating on the turn of events and advancement in the market utilizing digital marketing strategies—the chances for the globalization of digital marketing instruments all over the world. Digitalization of the economy and improvement in virtual space have become the fundamental elements of the economies of the central countries, which influence all circles of life. Expanding processes in the world requires new formats and adjusting the eyes on marketing (Brych et al., 2019).

The few studies composed that a lot of consideration has been centered around the huge chances digital advertising, with less consideration on the organizations, are confronting going digital. The studies have investigated the parts of promoting get new implications, and the new promoting correspondence channels offer better approaches for publicizing and new pointers to quantify the impacts (Kotane et al., 2019).

The digital change process covers four key business regions, advanced change, trade, content, local business area, and mutual effort. Other than promoting channel go-betweens, the digital change process influences the jobs and conduct of customers while doing their deals. It is composed that social advancements profoundly disturbed interchanges, promoting, and consumers (Kagermann, 2015). Digital marketing cooperates between consumers and suppliers by presenting computerized data, correspondence, and computerized advances. From a more extensive perspective, digital marketing is about promoting work with the execution of digital data, which figures on economic development. Digital marketing is viewed as an important piece of digital advertising, which is grown along with it.

Undoubtedly, the industrial revolution of the 21st century has left the whole world to experience a new era of economic development (i.e., digital marketing). Digital marketing has emerged as a fundamental development to the information revolution. The significant developments in the ICT have extended the scope of the nations by quickly adapting to the changing market needs and customer’s satisfaction. In particular, digital marketing has led to the new definition of marketing. Technological development has fostered the digitalization process, which makes that those digital enterprises reach the target customers. Indeed, the increasing importance of the ICT has radically enhanced the socioeconomic structure by making businesses to realize the importance of digital innovation and efficiency (Aydoğan, 2020).

H3: A significant relationship between rules and regulations and economic development was found.

### The Mediating Role of Digital Enterprise

Digital enterprise centers around the conversation of the improvement system of China’s digital economy and its correlation with different areas. In light of the fundamental structure of the digital enterprise, with the extending of digitalization, researchers accept that the mix of technological advancement and conventional businesses can accomplish and strong the digital enterprise: understand the green and fast advancement of the GDP, understand the change in the utilization structure, work on the nature of human resources, drive the digital economy from being work serious to becoming innovation concentrated, solidify framework development, make full and powerful utilization of information assets, the mechanical advancement, extend incorporated applications, and establish a casual setting to enable the digital enterprise (Gregory et al., 2019). In the new digital economy, innovation is the prime component driving the firms’ technical progress, thus contributing toward economic development. The growing e-commerce application fosters firms’ digital activities, thereby strengthening the impact of e-commerce digitalization on global economic advancement.

Accordingly, China ought to benefit from regions such as 5G, in advanced worldwide rivalry, further reinforcing key center advances’ innovative work and fostering the entire business chain. Different researchers trust that there are a few issues in the advancement of China’s digital enterprise, like the lopsided, insufficient, and ungraceful turn of events. These issues are primarily moved in the spaces including digital marketing, the level of redesigning of the advanced business, data network security, and the e-commerce sites and digitalization (Payne and Frow, 2004).

China’s economy is in a time of the momentary turn of events and slow development. The improvement of the economy relies more upon the quality and effectiveness of monetary development than on amount and speed with the relationship among digitalization, digital marketing, and e-commerce. The quantitative signs of monetary development, such as GDP and public pay, are as of now not the main focal point of the public development, as the center has steadily moved from amount to quality to advance the development of assets of digital enterprises of China (Zhu and Kraemer, 2005).

Beginning with the e-commerce development of financial aspects, the business analysts have consistently seen the rise in profits such as the economy’s development. With the assistance of the econometric model, attention to that, there is critical aspects of e-commerce benefits in China’s digital enterprise. The economic development lies in buying selling goods on online platforms such as Ali-Express, Amazon, and eBay. The following hypothesis is stated:

H4: Mediation of digital enterprise exists between e-commerce and economic development.

To comprehend economic development, different researchers utilize different econometric models to investigate the particular impacts of driving variables such as digital marketing, political stability, direct investment, human resources, banking globalization and data, and correspondence innovation on the quantitative marks of economic development. By measuring digital marketing, promoting goods and service through online platforms and websites, a digital enterprise is here, which manage all functions to improve the economic development of any country (Duch-Brown et al., 2017).

In recent years, digital emergence has played a significant role in business marketing. With the evolution of e-commerce, marketing becomes increasingly essential for the development of firms. The establishment of digital enterprises mediates the relationship between digital marketing and economic development. Value creation through digital marketing supports the evolution of digitalization (Kumar et al., 2020), thus promoting economic advancement. Indeed, the digital revolution brought in the field of marketing has revolutionized consumer’s behavior by strengthening the importance of the ICT, which has significantly changed the concept of traditional marketing (Shpak et al., 2020). Digital marketing has helped marketers to achieve enduring advantages, thus improving firms’ economic performance.

Fundamentally, technology holds immense potential for organizations in the shape of higher financial returns. With respect to investigating the connection between digitalization and great financial turn of events, more researchers have concentrated on the effect of the digital enterprise on improvement according to the viewpoints of enormous information strengthening, the coordination of the digital enterprise and the economic development, shared economy, digital money, and strategy supply frameworks (Barua et al., 2001). For the most part, the advanced economy is following the new improvement idea of development, coordination, green, receptiveness, and sharing. China is turning into a significant vital improvement course, which will advance China’s economic development.

The above research results show that the studies of researchers in the digital enterprise are generally broad, which include the meaning, attributes, sway impacts, record framework development and assessment, and different angles. There are not many observational examinations on the advancement of economic development by the digital enterprise, and to concentrate on the unique changes of the advancement of the digital enterprise and its impact on the economic development of China, which prompts the restrictions of the assessment of the file framework and the absence of congruity in the perception of the improvement in a digital enterprise (Hornby et al., 2002).

Based on past studies of the digital economy, this paper chooses the center aspects and relationship of the digital economy. It attempts to take the improvement in the digital enterprise in China as the exploration object to assess the general changes and provincial contrasts in improving China’s economic development. An econometric model is built to concentrate on the impact of the digital enterprise on the great financial turn of events. Economic development is presented as a delegate variable between the two angles; growing in this manner, an investigation of the impact of the digital enterprise to sustain consistency on the monetary turn of events is measured according to another point of view (Julian and Ahmed, 2005).

Furthermore, this paper likewise inspects the cooperation between the digital enterprise and economic development to concentrate on the digital economy in advancing excellent financial turn of events. Also, this study clarifies the effect of the digital enterprise on economic development with e-commerce, digital marketing, and digitalization. The discoveries have great approach reference on incentive for China in endeavors to speed up the advancement of the digital enterprise (Al-Hyari et al., 2012).

H5: Mediation of digital enterprise exists between digital marketing and economic development.
H6: Mediation of digital enterprise exists between digitalization and economic development.
H7: A significant relationship exists between digital enterprise and economic development.

### The Moderating Role of Sustainability

Sustainability models of economic development are progressively becoming more appropriate from digital enterprise and their factors of e-commerce, digital marketing, and digitalization in countries worldwide. The digital enterprise gives another impulse and course for the management of economic development. Focus on the “digital enterprise” showed up in a report by the Chinese government. Additionally, a report from 2019 proposed to “increase the development of another digital economy and industry groups, and significant relationship with digital enterprise and economic development” (Kshetri, 2007).

The studies show the need to extend the commitment of “digital enterprise and concerning the advancement of the economic development with the moderation of sustainability. The public, financial, and social advancement of the People of China and the layout of long-term objectives for 2035” and an objective to expand the additional value of China’s digital economy and businesses (Lawrence and Tar, 2010). The previously mentioned studies show that it has a significant relationship to digital enterprise and economic improvement.

The digital enterprise has an impact on economic developing areas. Maintaining proper economic development and sustainability is the financial and social turn of events. The responses to these inquiries depend on the foundation of the sustainability framework for economic development. Research on the digital enterprise is as yet in its early stages; its connection, characterization, and estimation. In this way, there are somewhat not many hypothetical investigations and studies on the digital enterprising and their events and economic development.

H8: A significant moderation of sustainability exists between digital enterprise and economic development.

**FIGURE 1:**
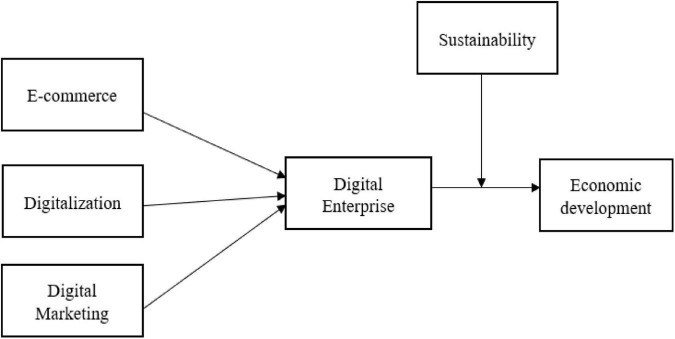
Conceptual framework.

Economic suitability states the production and consumption with regard to future needs. The sustainability theory supports economic development and economic relief of the country. The study states that sustainability with economic development shows the technological advancements and perceives natural resources which sustain economic development. The developing size of the financial framework has stressed the normal asset base. An economic development considering the hypothesis of “monetary maintainability” is obliged by the prerequisites of “natural supportability.” It controls assets to guarantee the “maintainability” of normal capital. It has become ordinary to call for replacing the predominant regulation of economic development with another precept of financial development for seeking after a type of subjective development rather than economic development (Ruttan, 1991). Figure 1 shows the study theoretical framework.

## Methodology

For analyses of the adopted variables and concepts, this section encourages using appropriate tools and techniques for analyzing the relationship among digitalization and economic development. The study has followed a quantitative approach and conducted surveys for data collection, and it has mainly focused on standards and statistics. It has used numerical values through questionnaire surveys and measured through SPSS and SmartPLS. The software (i.e., SPSS and SmartPLS) had brought colossal benefits that have helped the researchers to develop the analysis section. The SPSS is a statistical software tool that helps forecast future trends, whereas SmartPLS is a graphical tool that assists in recording the study results through regression algorithms. Perhaps, both the tools had enabled us to evaluate the data, thereby providing valuable results.

Accordingly, for this study, the data were collected from China customers. The data collection technique is primary, in which researchers have used survey analysis. The researcher has sued the purposive sampling technique to collect data. Purposive sampling adopted for data gathering helps to record the participant’s responses. This non-probability sampling technique allowed us to choose the participant for the study survey. The objective of the study is to determine the mediating role of the digital enterprise, e-commerce, digitalization, digital marketing, and economic development. In support, this sampling technique helped in selecting the Chinese’ customers as the sample for achieving the study objective. Given this, the study had floated 480 questionnaires, and out of that, 400 valid questionnaires were received for data analysis. A sample of 120 respondents led the pilot study to check the reliability of the items. Further, the data have been processed through SPSS and SmartPLS.

The scale for the construct of e-commerce consisted of three items and was adapted from the study of Saeed et al. (2005). The digitalization scale consisted of three items and was taken from the study of Meroño-Cerdan and Soto-Acosta (2005). The scale for digital marketing is taken from the study of Chen and Zhang (2015), and it has three items. In this study, Hu et al. (2011)’s three items scale of digital enterprise was adopted. Beheshti and Salehi-Sangari (2007)’s three items scale was adopted to measure the sustainability variable. Pechlaner et al. (2014)’s three items scale was adopted to measure economic development.

## Results

This study has used the structural equation modeling in the SmartPLS 3.3 latest version. Structure equation modeling (SEM) is used to measure the relationship among dependent, independent, and mediator. It is the most used method to measure the path coefficients. The construct has three independent variables: a mediator, a moderator, and a dependent variable. The SEM consists of two steps of analysis PLS algorithms and bootstrapping. PLS algorithms are the weighted vector-based regression analysis model, which shows coefficient values. Regression models generate from bootstrapping values. The total number of respondents was 400; 180 (45%) respondents were women, and 220 (55%) respondents were men. The education of the respondents is as follows: 60 respondents have diplomas (15%); among them, 65 (16.25%) had bachelor’s degrees, 85 (21.25%) had master’s degrees, 90 (22.5%) had MPhil., and 100 (25%) had Ph.D.

### Measurement Model Assessment

The measurement model has been analyzed in SmartPLS, and it shows composite reliability of the variables, average variance extracted values, and their factor loadings. The Cronbach’s alpha values show the reliability and validity of the data and their consistency of scales. The average variance extracted (AVE) values are collected throughout the study and the quantity of variance in the statistical hypothesis. AVE values should be greater than 0.5; if any value is lower than 0.5, its item can be deleted or changed according to the study requirement.

The composite reliability (CR) values should be greater than 0.7, which shows the reliability and consistency of the data. AVE should be greater than 0.5, and CR value should be greater than 0.5 of one variable. It shows a high significance level. It ensures the threshold level of the study among sectors. The next step of the study is to check the discriminant validity of the data, which involves a few steps. Cross-loadings, heterotrait–monotrait (HTMT) ratio, and factor loading occur when one factor depends on more than other factors (Fornell and Larcker, 1981). It reflects the dependency of the data.

Heterotrait–monotrait ratios show the correlation among variables; its range is −1 to + 1. It should be less than one, and it considers a strong relationship between two variables at a significance level of 0.01. Furthermore, this study has also measured effect size F- and R-square, which shows the significance and dependency of the data. The acceptable range of R-square is from 0.3 to 0.7. Cross-loadings, HTMT ratio, and factor loading occur when one factor depends on more than other factors (Fornell and Larcker, 1981). It reflects the dependency of the data. Table 1 shows the study measurement model.

**TABLE 1:** Measurement model.

	Items	Loadings	Cronbach’s alpha	rho_A	CR	AVE
E-commerce	EC1	0.762	0.787	0.815	0.798	0.610
	EC2	0.816				
	EC3	0.772				
Digitalization	DIG1	0.817	0.813	0.818	0.767	0.663
	DIG2	0.709				
	DIG3	0.767				
Digital marketing	DM1	0.716	0.872	0.893	0.788	0.718
	DM2	0.890				
	DM3	0.811				
Economic development	ED1	0.819	0.709	0.845	0.812	0.734
	ED2	0.798				
	ED4	0.890				
	ED5	0.682				
	ED6	0.907				
Sustainability	SUS1	0.712	0.882	0.784	0.873	0.716
	SUS2	0.784				
	SUS3	0.715				
Digital enterprise	DEP1	0.801	0.893	0.787	0.815	0.722
	DEP2	0.829				
	DEP3	0.861				

Table 2 shows Fornell–Larcker criterion values. The relationship between e-commerce and economic development is 0.672, which shows a strong relationship between them, and it is acceptable in the study above than acceptance criteria. The relationship between digitalization and economic development is 0.719, which has a solid and significant relationship between them, and it is more incredible than 0.5. The relationship between digital marketing and economic development is 0.679, which shows a strong relationship. The relationship between digital enterprise and economic development is 0.525, which shows a strong relationship between them. Sustainability has 0.711 values, which has a solid and significant relationship between them.

**TABLE 2:** Fornell–Larcker criterion.

	EC	DIG	DIM	DEP	SUS	ED
E-commerce	0.504					
Digitalization	0.581	0.505				
Digital marketing	0.459	0.518	0.632			
Digital enterprise	0.721	0.635	0.529	0.620		
Sustainability	0.490	0.720	0.711	0.669	0.711	
Economic development	0.672	0.719	0.679	0.525	0.741	0.572

Table 3 presents HTMT ratios, which show the correlation among variables; its range is −1 to + 1. It should be less than one, and it considers a strong relationship between two variables at a significance level of 0.01. Furthermore, this study has also measured effect size F- and R-square, which shows the significance and dependency of the data. The acceptable range of R-square is from 0.3 to 0.7.

**TABLE 3:** Heterotrait–monotrait (HTMT).

	EC	DIG	DIM	DEP	SUS	ED
E-commerce						
Digitalization	0.418					
Digital marketing	0.605	0.635				
Digital enterprise	0.503	0.690	0.457			
Sustainability	0.516	0.465	0.562	0.615		
Economic development	0.711	0.513	0.718	0.506	0.617	

Using the HTMT as a criterion involves comparing it to a predefined threshold. E-commerce and economic development have 0.711 values, which state the relationship between them. Digitalization has a positive and significant relationship of 0.513 with economic development. Digital marketing has a positive and significant relationship with the economic development of 0.718, which lies within the criterion region. Digital enterprise has 0.506 values that show a strong relationship with economic development. Sustainability has 0.617 values that show a strong relationship with economic development.

### Structural Model

**FIGURE 2:**
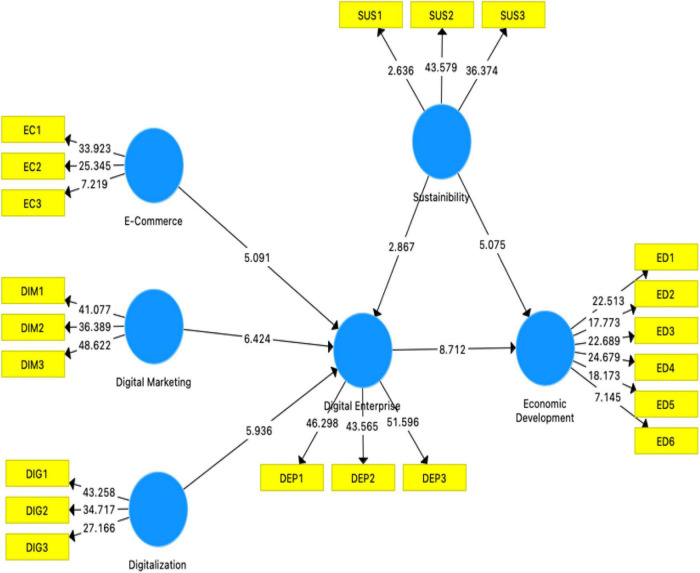
Structural model results.

The study structural model shows path coefficient values, which are beta value, *t*-value, *p*-value, standard error, and LLCI and ULCI (see Figure 2). The bootstrapping shows a significant value of *p*-value, which states acceptance and rejection of the hypothesis. Based on this study criteria, all hypotheses are accepted and supported as *p*-values are significant and *t*-values are greater than 1.96. All hypotheses are accepted and significantly found a relationship between them, and all hypotheses are supported. Table 4 shows the impact between digitalization support and digital enterprise (*t*-value = 2.182, *p*-value = 0.001). The hypothesis is accepted and supported as e-commerce has a significant positive relationship with digital enterprise (*t* = 13.001, *p* = 0.000). Same as digital marketing has a positive and significant relationship with digital enterprise (*t* = 15.232, *p* = 0.000). The table below shows the impact between e-commerce and economic development (*t* = 3.161, *p* = 0.021). The hypothesis is accepted and supported as e-commerce has a significant positive relationship with digital enterprise (*t* = 12.139, *p* = 0.000). Digital marketing has a positive and significant relationship with digital enterprise (*t* = 6.719, *p* = 0.000).

**TABLE 4:** Hypothesis results.

Hypothesis	Std Beta	SD	*t*-values	*p*-values	ULCI	LLCI
Digitalization support— > Digital enterprise	0.311	0.221	2.182	0.000	0.112	0.435
E-commerce— > Digital Enterprise	0.243	0.325	13.001	0.000	0.101	0.411
Digital marketing — > Digital enterprise	0.232	0.312	15.232	0.000	0.103	0.315
Digitalization support— > Economic development	0.182	0.167	3.161	0.001	0.113	0.308
E-commerce— > Economic development	0.186	0.176	12.139	0.000	0.181	0.417
Digital marketing — > Economic development	0.189	0.187	6.719	0.000	0.141	0.328
Digitalization support— > Digital enterprise— > Economic development	0.182	0.125	12.081	0.000	0.121	0.232
E-commerce— > Digital enterprise— > Economic development	0.152	0.189	17.061	0.001	0.115	0.406
Digital marketing— > Digital enterprise— > Economic development	0.174	0.128	5.812	0.000	0.122	0.372
Digital enterprise— > Economic development	0.243	0.172	4.101	0.000	0.116	0.215
Political support— > Sustainability— > Economic development	0.281	0.177	3.872	0.001	0.122	0.256

The mediation of digital enterprise has a significant relationship between digital marketing and economic development (*t* = 12.081, *p* = 0.000). The mediation of digital enterprise has a significant relationship between e-commerce and economic development (*t* = 17.061, *p* = 0.001). The mediation of digital enterprise has a significant relationship between digital marketing and economic development (*t* = 5.812, *p* = 0.000). Also, digital enterprise has a significant relationship with economic development (*t* = 4.101, *p* = 0.000). The moderation of sustainability exists between digital enterprise and economic development (*t* = 3.782, *p* = 0.000).

## Discussion

In today’s world of globalization, the transition of conventional business practices to e-commerce businesses has led the world to relish the colossal benefits of the information age. In the current period of internationalization, digitalization has overwhelmed global business activities, which makes the economic factors accelerate the business processes. The developing technologies have made the business adopt innovative techniques, boosting worldwide economic development. In particular, these accelerating trends have tremendously contributed toward achieving global sustainable development (Liu et al., 2018).

Essentially, the increasing economic development in the digital enterprise has received considerable attention in the last few decades. Digital enterprises have largely improvised their productivity level, thus promoting economic advancement (Intrieri et al., 2018). The increasing functions of digital enterprises have significantly modernized the country’s economic structure, thereby reducing the cost of efficiency and innovation. In particular, this digital transition has improved the quality and development of the economic transformation, thereby accelerating the firms’ growth. Given this, the study analysis shows that e-commerce and digitalization have a significant positive impact on economic development. Digitalization is an integrated network that provides immense benefits to organizations, society, and countries. The emerging information technologies (i.e., ICTs) transform business activities, reduce the ICT cost, facilitate information access, and enable firms’ to achieve economic continuality (Palvia et al., 2018).

Consistently, the transformative effect of ICTs fosters firms’ economic growth, with digital marketing playing a fundamental role in gaining business prosperity. Digital innovative marketing has contributed toward achieving economic progression by meeting the diverse needs of potential customers. The rapid digital growth in marketing has changed the way of commerce across borders, subsequently ensuring socioeconomic stability (Aydoğan, 2020). In support, the study results have been found consistent with the prior literature, which indicated digital marketing as a prominent tool driving the firms’ economic activities (Başyazicioğlu and Karamustafa, 2018).

In particular, the study shows that digital transformation has a significant positive impact on firms’ sustainability and growth (El Hilali et al., 2020). The digital transformation ensures organizations’ sustainability by radically enhancing the firms’ innovation process. The digital enterprise capability makes the businesses transform their marketing activities by implementing modern digitalized tools that allow firms to achieve sustainable development (Gil-Gomez et al., 2020). This digital transformation continuously ensures that the company delivers better customer service than the competitors. The digital marketing media manifest the incorporation of digital technologies in fostering economic progress. Perhaps, to survive in the competitive market, digital marketing is a valuable phenomenon driving the business operations toward sustainability growth (Duffett et al., 2019). According to the data results and findings, the study has found a significant impact between variables, and all hypotheses are accepted. The study contributes toward digital enterprise and is moderated by sustainability by measuring the impact of digitalization support, economic development, and digital marketing on economic development. The change that accompanies growth is natural, and most of it is welcome, but providing some mechanisms to protect the company’s identity and serve as some guidance for renewal and change is necessary. The corporate brand may present such a mechanism.

The study has found a significant and positive relationship as *t*-values are greater than 1.96, and all hypotheses are supported. The study found a significant mediating relationship of digital enterprise among digitalization support, economic development, and digital marketing on economic development, and findings are consistent with the study of Tesfom and Lutz (2006). Same as it is found that moderation of sustainability exists between digital enterprise and economic development, and the *t*-value is greater than 1.96, and *p*-values are significant. It has increased the country’s digital enterprise in China (Ferguson et al., 2005). All the findings are consistent with previous studies.

The consequences of the assessment affirmed that political shakiness (both inward and outside) altogether affects monetary execution. Precisely, the paper has verified the view that inward political flimsiness, as proxied by the quantity of CR, adversely affects financial execution. These outcomes are reliable with past local and global investigations (Van der Eng, 2006). Further, the review uncovered the consequence of the level of opportunity on China’s economic development. At last, the examination has also shown a positive effect of outside digital enterprise on economic development, which has been credited to the development of capital (both physical and human) and worldwide help.

## Conclusion

This review examined the effect of e-commerce, digitalization, and digital marketing on economic development with the mediation of digital enterprise. The study covered that e-commerce, digitalization, and digital marketing use with GDP per capita had a since quite a while ago run sway dependent on the test results; additionally, all e-commerce, digitalization, and digital marketing consumption were found to decidedly affect economic development, yet e-commerce, digitalization, and digital marketing had a more grounded improvement upgrading impact. Likewise, different factors such as government size and well-being consumption also affected GDP per capita.

Along these lines, the approach this review suggests is that in view of the significance of e-commerce, digitalization, and digital marketing in the monetary turn of events and social government assistance, states ought to take on proper strategies and give the essential conditions to the turn of events and economic development. For this reason, as per the discoveries of exact exploration, it is suggested that the public authority gives further consideration to monetary preparation to develop further e-commerce, digitalization, and digital marketing, and mediation of digital enterprise, and it could ultimately prompt financial advancement in the country.

### Theoretical Implications

This review examines the effect of digital enterprise on economic development for country-explicitly China. This cross-sectional study also examines GDP development’s subareas such as horticulture development, modern, and administrations. Economic development has so many economic, social, and political divisions. Digital Enterprise has many other factors that are not taken in the study. It adds to the literature; digital enterprise affects economic development for China alongside macroeconomic factors.

The primary commitment of the review is to research the effect of digital enterprise on the subarea development of China. The outcomes show that digital enterprise emphatically affects GDP and subarea development in China because political dependability upgrades the nature of administration and works on the nature of law and order. Economic development decidedly affects financial development and subsectors of GDP aside from modern area development. This outcome shows that economic development is so high because of arrangements by the political foundations. Economic development improvement affects the most extraordinary outcomes against farming development in China.

### Practical Implications

This study shows that the developmental area is less beneficial for the economy for many reasons. Economic development moves quicker with the assistance of digital enterprise on markets. An economic development area has sustainability for advancing and economic development. This study concentrates on experimentally digital enterprise areas, the idea that economic development with digital enterprise is the element that leads to economic development. It ought to work on digital enterprise and improve the country’s credibility. Mainly, it is stated with developments and a more impressive and maintainable digital enterprise that can help the exercises for practical economic development.

Furthermore, digital enterprise plays a fundamental role in the development of society. The study suggests that the digital economy cultivates entrepreneurial opportunities through accelerating innovative ideas and information. It aims to meet diverse customer needs, thus promoting economic growth. Hence, to achieve socioeconomic sustainability, the researchers should ensure the continuous widespread of knowledge and information for promoting the establishment of digital enterprises in developing countries. This innovative thinking makes the entrepreneur to realize the significance of digital technologies, thereby accelerating new momentum to innovation-driven economic development. In particular, the digital revolution technologies ensure the development of healthy social welfare.

### Limitations and Future Work

It desires to additionally talk about the disintegration of digital enterprise exchanges into deals and obtainment (e-commerce, digitalization, and digital marketing), alongside the connection between information factors, at the point when more itemized information would open up. Likewise, it would be intriguing to recognize various channels (decrease the exchange cost among purchasers and dealers or solid productivity improvement in the creation and supply of chain processes) through which digital enterprise can raise GDP and improve economic development.

## Data Availability Statement

The raw data supporting the conclusions of this article will be made available by the authors, without undue reservation.

## Ethics Statement

Ethical review and approval was not required for the study on human participants in accordance with the local legislation and institutional requirements. Written informed consent for participation was not required for this study in accordance with the national legislation and the institutional requirements.

## Author Contributions

The author confirms being the sole contributor of this work and has approved it for publication.

## Conflict of Interest

The author declares that the research was conducted in the absence of any commercial or financial relationships that could be construed as a potential conflict of interest.

## Publisher’s Note

All claims expressed in this article are solely those of the authors and do not necessarily represent those of their affiliated organizations, or those of the publisher, the editors and the reviewers. Any product that may be evaluated in this article, or claim that may be made by its manufacturer, is not guaranteed or endorsed by the publisher.
